# Perceptions of telemedicine and teledermatology among safety net patients, health care professionals, and clinical support staff

**DOI:** 10.1016/j.jdin.2026.07.008

**Published:** 2026-07-30

**Authors:** Adam Cardenas, Mir Rabby, Ashley Koszarek, Karen Jimenez, Arely Ibarra, Adewole S. Adamson, Lia E. Gracey

**Affiliations:** a Dell Medical School, The University of Texas at Austin, Austin, Texas; b Moody College of Communications, The University of Texas at Austin, Austin, Texas; c Pediatric and Adolescent Dermatology, Dell Children's Medical Group, Austin, Texas; d Division of Dermatology, Department of Internal Medicine, Dell Medical School, The University of Texas at Austin, Austin, Texas; e CommUnityCare Health Centers, Austin, Texas

**Keywords:** electronic, health services research, internet, practice, technology, teledermatology, telemedicine

Telemedicine and teledermatology allow patients to receive their health care remotely; both have been understudied in safety net health care settings. Our objective was to explore the perceptions of telemedicine and teledermatology among key stakeholders at federally qualified health centers, including patients, health care professionals (HCPs) (eg, physicians and nonphysician providers), and clinical support staff (CSS) (eg, nurses and medical assistants).

Our study used English/Spanish surveys developed from a literature and resource review,^1^, ^2^, ^3^, ^4^, ^5^ piloted with 21 participants excluded from analysis and analyzed with descriptive and inferential statistics (eg, *t* tests), alongside a thematic analysis of interviews using grounded theory. Cronbach α reliability tests were conducted to determine internal consistency. Convenience sampling was used for patients and CSS (100% response rate from medical assistants), including fliers, in-person tabling, and an email listserv with HCPs (18.1% response rate). Patient and other CSS response rates were unable to be calculated as participants voluntarily came to research tables or replied to fliers. Data were collected from June 2024 to June 2025 at federally qualified health centers in Austin, Texas. This project received exemption determination by the institutional board at University of Texas at Austin (IRB STUDY00005479).

One hundred and seventy-eight individuals completed the survey, including 79 patients, 20 HCPs, and 79 CSS (Supplementary Tables 1 to 3, available via Mendeley at https://data.mendeley.com/datasets/mn4g9mk9xf/2). Among patients, 77.2% indicated Hispanic, Latino, or Spanish origin, along with 66.7% of HCPs and CSS. Additionally, 59.5% of patients reported <$30,000 household income. HCPs (mean [M] = 4.25, SD = 0.72) showed significantly higher positive perceptions of telemedicine safety/security than patients (M = 3.84, SD = 1.13, *t*(45.83) = −2.03, *P* = .048). However, patients who had used telemedicine before (M = 4.18, SD = 0.89) reported significantly more favorable safety perceptions of telemedicine compared with those who had not ([M = 3.50, SD = 1.24], *t*[70.59] = 2.81, *P* = .006). Among patients, 57 (72.1%) reported reliable access to the internet at home and 56 (70.9%) patients felt that they could use a computer or smartphone to send photos for their health care to their doctor (Table I). Fifteen (75%) HCPs either strongly or somewhat agreed that the time to send a teledermatology consultation to a dermatologist is reasonable.

**Table 1:** Survey results among patients regarding telemedicine and teledermatology

**Question**	**No. (%)**
How often do you use a desktop computer or laptop?	
Every day	12 (15.2)
A few times a week	18 (22.8)
A few times a month	7 (8.9)
A few times a year	9 (11.4)
Rarely or never	28 (35.4)
I don't know	5 (6.33)
How frequently do you use the internet to access healthcare information or services?	
Often	33 (41.8)
Sometimes	33 (41.8)
Rarely	9 (11.4)
Never	4 (5.1)
Have you ever used telemedicine as part of your healthcare?	
Yes	39 (49.4)
No	40 (50.6)
I have reliable access to the internet at home.	
Strongly agree	37 (46.8)
Somewhat agree	20 (25.3)
Neither agree nor disagree	8 (10.1)
Somewhat disagree	4 (5.06)
Strong disagree	10 (12.7)
I believe telemedicine is safe and secure.	
Strongly agree	25 (31.6)
Somewhat agree	31 (39.2)
Neither agree nor disagree	12 (15.2)
Somewhat disagree	7 (8.9)
Strong disagree	4 (5.1)
I can use a computer or smartphone to have a virtual doctor’s appointment for my healthcare from home.	
Strongly agree	30 (38.0)
Somewhat agree	25 (31.6)
Neither agree nor disagree	5 (6.3)
Somewhat disagree	10 (12.7)
Strong disagree	9 (11.4)
I can use a computer or smartphone to send photos for my health care to my doctor.	
Strongly agree	33 (41.8)
Somewhat agree	23 (29.1)
Neither agree nor disagree	5 (6.3)
Somewhat disagree	6 (7.6)
Strong disagree	12 (15.2)

Thirty-four individuals were interviewed (23 patients and 11 HCPs and CSS), with 14 (61%) patient interviews in English and 9 (39%) in Spanish. Overarching themes related to telemedicine for all groups included barriers, convenience, human connection, no barriers, and quality of care (Table II). Among patients, the most frequent theme was a desire for a human connection (28 instances); notably, this was not as frequent a theme among HCPs.

**Table II:** Qualitative analysis of telemedicine interview discussion among patients, health care professionals, and clinical support staff

Theme	Codes	Exemplar quotes
1. Barriers		
Frequency: Patients: 22 Professionals and staff: 11 Total: 33	Access to technology	Patient: “I had trouble connecting one time, but that was just like there was something wrong and buggy with the software.”
		Health care professional: “They [patients] don't have access maybe to a phone that has video capability.”
	Technical literacy	Patient: “I'm not good with computers. Anything so….If my phone is on mute or something. I don't love it.”
		Clinical support staff: “The patients are not that tech savvy. So the video one, like we'll have a video visit, but it always turns out to be just phone.”
	Other access	Patient: “No insurance, can't afford them (telemedicine visits).”
		Health care professional: “Either because there's a language barrier or there's a financial barrier.”
	No other option offered	Patient: “They’ve never offered me a consultation by phone, although that would be good too.”
		Health care professional/clinical support staff: N/a
2. Convenience		
Frequency: Patients: 23 Professionals and staff: 21 Total: 44	Accessibility	Patient: “It's nice, because I don't have to drive all the way there. Look for parking, or get stressed about making my visit. I can just do it when it's convenient. I can usually do it at work.”
		Health care professional: “It gets them access to a specialty (dermatology) that would generally take like 2 or 3 months for them to get in.”
3. Human connection		
Frequency: Patients: 28 Professionals and staff: 12 Total: 40	Being understood	Patient: “I have the feeling that I could not express myself as I wanted and that the person could not understand my emotions as I needed to express them.”
		Health care professional: “It wasn't by any fault of the telemedicine for the one that didn't go very smoothly. It was more like it was an older gentleman who had some health literacy issues. So he we was trying to like explain to us what medications he took, and we really probably just need to see them in person.”
	Preference for in-person care	Patient: “I don't know that I would feel as confident with telemedicine should I need some sort of like physical examination, or like, you know, would want a doctor to actually see something that is bothering me on my body.”
		Health care professional: “Half of the benefit of going to see the doctor, is people almost use as like therapy sessions… sometimes there's like a human connection I think is really valuable.”
	Visual learning	Patient: “I am very visual. So when I talk to people, I need to see them face to face.”
		Health care professional: “I am not huge fan of telephone, though, um, especially, like, with my psych patients because I like to see (their face).”
	Nonverbal cue needs	Patient: “There's not going to be that humanity, that eye contact.”
		Health care professional: “It's really easy to say, you know, “I'm feeling fine,” but until you really can, like, see them and talk to them a little bit more, um, telephone, I think, is a lot harder.”
4. No barriers		
Frequency: Patients: 3 Professionals and staff: 1 Total: 4	Lack of barriers	Patient: “I have health insurance that covers telemedicine appointments. So for me personally, it's incredibly easy to access telemedicine.”
		Clinical support staff: “I think it's much better for our patients to be able to have access to televisits, just because I mean, not everybody can get here.”
5. Quality of care		
Frequency: Patients: 9 Professionals and staff: 7 Total: 16	Appropriateness	Patient: “I see a couple of doctors through telemedicine, neither of which really require any sort of physical examination. And I've had no issues with it.”
		Health care professional: “If it's…like an acute concern, something that, like, you know, would require like a more in depth exam like, then they're less able to utilize our time on the videos.”
	Clinical limits	Patient: “Well, if I go to the doctor to be seen, they have to listen to my lungs or for example, if I feel that my throat is sick then they will need to check my throat. But through a video call, they can't do that.”
		Health care professional: “except we're in between a screen and that's a lot of the issue with the telemedicine is, you know, if they're doing a blood pressure follow-up, sometimes they don't have a blood pressure machine and it's like, “Well how can I accurately follow up on your blood pressure if you don't have a machine?”
	Follow-up use	Patient: “The postpartum of like right after I had my baby, like the 2 week follow up that they do, they just automatically did a follow up instead of like coming in. And that's just easier. Because after C section, right? Trying to get the baby, and you know. So that was easier just to come on the phone.”
		Health care professional: “Like a medication refill, or something like where they [patients]…have their blood glucose checks… we can go over it if we're going over labs.”

Our data suggested that patients have great concern about maintaining human connection with their physicians; technology access is not the primary concern. Our findings suggest that familiarity with telemedicine is associated with patients viewing remote care favorably; it underscores the need to promote telemedicine literacy among patients, especially in areas with fewer health care facilities. Teledermatology is viewed positively by HCPs with an accessible workflow in our federally qualified health center. Limitations of our study include single-center design, limited sample size, and selection bias with convenience sampling.

## Conflicts of interest

Dr Gracey is an associate editor for the International Journal of Women's Dermatology and is a member of the American Academy of Dermatology Teledermatology Committee. Dr Adamson serves as deputy and web editor for *JAMA Dermatology*. Authors Rabby, Koszarek, and Ibarra and Drs Cardenas and Jimenez have no conflicts of interest to declare.
